# Genome evolution in Reptilia: *in silico *chicken mapping of 12,000 BAC-end sequences from two reptiles and a basal bird

**DOI:** 10.1186/1471-2164-10-S2-S8

**Published:** 2009-07-14

**Authors:** Charles Chapus, Scott V Edwards

**Affiliations:** 1 Department of Organismic and Evolutionary Biology, Museum of Comparative Zoology, Harvard University, 26 Oxford Street, Cambridge, MA 02138, USA

## Abstract

**Background:**

With the publication of the draft chicken genome and the recent production of several BAC clone libraries from non-avian reptiles and birds, it is now possible to undertake more detailed comparative genomic studies in Reptilia. Of interest in particular are the genomic events that transformed the large, repeat-rich genomes of mammals and non-avian reptiles into the minimalist chicken genome. We have used paired BAC end sequences (BESs) from the American alligator (*Alligator mississippiensis*), painted turtle (*Chrysemys picta*) and emu (*Dromaius novaehollandiae*) to investigate patterns of sequence divergence, gene and retroelement content, and microsynteny between these species and chicken.

**Results:**

From a total of 11,967 curated BESs, we successfully mapped 725, 773 and 2597 sequences in alligator, turtle, and emu, respectively, to sites in the draft chicken genome using a stringent BLAST protocol. Most commonly, sequences mapped to a single site in the chicken genome. Of 1675, 1828 and 2936 paired BESs obtained for alligator, turtle, and emu, respectively, a total of 34 (alligator, 2%), 24 (turtle, 1.3%) and 479 (emu, 16.3%) pairs were found to map with high confidence and in the correct orientation and with BAC-sized intermarker distances to single chicken chromosomes, including 25 such paired hits in emu mapping to the chicken Z chromosome. By determining the insert sizes of a subset of BAC clones from these three species, we also found a significant correlation between the intermarker distance in alligator and turtle and in chicken, with slopes as expected on the basis of the ratio of the genome sizes.

**Conclusion:**

Our results suggest that a large number of small-scale chromosomal rearrangements and deletions in the lineage leading to chicken have drastically reduced the number of detected syntenies observed between the chicken and alligator, turtle, and emu genomes and imply that small deletions occurring widely throughout the genomes of reptilian and avian ancestors led to the ~50% reduction in genome size observed in birds compared to reptiles. We have also mapped and identified likely gene regions in hundreds of new BAC clones from these species.

## Background

Knowledge of structural changes occurring in amniote genomes is critical for an understanding of patterns of genome evolution and of the evolution of species in general. Structural changes include deletion and insertion of coding or non-coding sequences, segmental translocations, and transposition. Structural variations in genomes are likely to underlie significant functional differences between species. Studies of the occurrence of such structural transformations between chromosomes can also permit reconstruction of genome structure of common ancestors. For example, many comparative studies have been performed in the mammals, including mouse/human comparisons [1,2] and human/chimpanzee comparisons [3]. These studies require the availability of a large amount of molecular data (complete or partial genome sequence, or BAC/YAC sequences). With the publication of the chicken genome [4,5], it is now possible to investigate structural relationships between the genomes of non-mammalian amniote species by comparative analyses.

Among the amniotes, the genome structures of reptilian species are poorly known, although studies in this group are increasing. Broad-scale synteny of whole chromosomes has been established between the chicken Z chromosome and chromosome 5 of turtle [6]. Recently, Kawai et al. [7] showed that the chicken Z exhibits synteny with various arms of autosomes in a turtle, crocodilian, and snake. However these studies, which have been based primarily on fluorescence in situ hybridization (FISH) analyses of BAC probes to chromosome spreads or whole-genome comparative hybridization [8,9], are still limited in taxonomic scope. Such chromosomal studies, as well as traditional molecular phylogenetic studies and global analyses of genome characteristics, can enlighten the phylogenetic relationships of species, for example the position of turtles, which is now generally agreed to fall near if not sister to the archosaur clade consisting of birds and crocodilians [10-15].

Because of their streamlined genomes, birds are excellent lineages taxa in which to conduct comparative and structural evolutionary genomics [16]. The chicken (*Gallus gallus*) is the closest species to non-avian reptiles for which the genome has been sequenced [5]. The size of the chicken genome size (1C) is 1.25 Gb. The emu (*Dromaius novaehollandiae*) is a ratite, belonging to the palaeognathous birds, a basal avian group [17]. The genome of the emu is 1.63 Gb [18-20] and is distributed among 40 chromosomes (1*n *= 1*x *= 40). Unlike Z and W sex chromosomes of the chicken, which are highly diverged, the sex chromosomes of the emu exhibit little dimorphism and exhibit evolutionary dynamics similar to emu autosomes [21]. The number of macro- and microchromosomes in the ratites is very similar to the chicken [21].

The genomes sizes of the American alligator (*Alligator mississipiensis*; 2.49 Gb) and the painted turtle (*Chrysemys picta*; 2.57 Gb) are roughly double that of chicken [22,23]. These species do not have dimorphic sex chromosomes – in fact, both species exhibit temperature-dependent sex determination (TSD) [24]. The American alligator karyotype is composed of 16 chromosomes, with no microchromosomes as in the chicken and the painted turtle [25]. The Emydidae, the family to which the painted turtle belongs, has a karyotype composed of 25 or 26 pairs of chromosomes (12–14 pairs of macrochromosomes and 12–14 pairs of microchromosomes) [26].

With their large insert sizes and ability to provide access to coding as well as noncoding regions, bacterial artificial chromosome (BAC) libraries provide another means of probing structural evolutionary changes in genomes [27,28]. Sequences of BAC clones have been used frequently to perform comparative studies such as construction of shotgun contigs, analyses of copy number variants and of physical maps, FISH mapping between species, identification of genes involved in diseases or building of virtual genomes [2,29-36]. BAC end sequences (BESs) are single-pass sequences obtained from each end of a BAC clones. These end sequences can be very specific markers and are excellent sources of sequence information that can be utilized in comparative genomics studies or for identification of orthologous regions between species [37]. In addition, because they ostensibly represent random snapshots of a given genome, BESs can be used to access genome content, repeated elements, protein-coding and conserved noncoding regions of a genome. For example, Shedlock et al [38] amassed several thousand BESs amounting to over 5 Mb of sequences from two reptile BAC libraries (American Alligator alligator and Painted painted Turtle) and used these sequences to study the distribution of repeat elements and microsatellites in these reptile genomes. Moreover, they documented some of the genomic differences that underlie the disparities in genome size between non-avian reptiles and birds. BESs are also useful means to develop phylogenomic markers. Thomson et al. [39] recently used several megabases of BESs from the painted turtle to develop a suite of markers that they used to examine rates of evolution and depths of taxonomic coverage.

To better understand structural evolution in the Reptilia (birds plus non-avian reptiles), we used BLAST to align BAC end sequences from American alligator, painted turtle, and emu with the chicken genome sequence. By mapping these sequences and studying their orientation in the chicken genome, we were able to document some of the types of changes that have accompanied the drastic difference in genome size between non-avian reptiles and birds.

## Materials and methods

### Databases

The BAC libraries from the alligator, turtle and emu were developed previously [40] and are available through the BAC clone program of SymBio Corporation http://www.sym-bio.com/. Each library is arrayed in 384-well plates and offers a high level of genome coverage for each target species (9.0×/11.2×/12.9×, respectively for alligator, turtle and emu). The alligator and turtle data were the same as those analyzed by Shedlock et al. [38] and were re analyzed for this study. For these species, five plates consisting of 1,675 clones were randomly chosen from their respective BAC libraries, clones were isolated, and both ends of each selected BAC clone were sequenced using the dideoxy method [38,40]. The total yield for alligator was 3,218 successful BESs (1543 BAC clones with both sequenced ends and 132 clones with only one end) with an average length of 770 bp and a total length of 2.5 Mb. For turtle, 3,461 BESs (1633 clones with both ends and 195 clones with only one end) were obtained (avg. length 703 bp, total combined length 2.4 Mb). For the emu, eight randomly chosen plates (total 2,936 clones) were subjected to end sequencing, yielding a total of 5288 quality reads (avg. length 662 bp, total combined length 3.5 Mb, 2352 clones with both ends and 584 clones with only one sequenced end). The alligator and turtle BES reads were generated at The Institute for Genomic Research, Rockville, MD http://www.tigr.org and the emu BESs at the Broad Institute, Cambridge, MA http://www.broad.mit.edu using published protocols [2,27]. All sequences were processed with Phred [41] and CROSS_MATCH to remove poor quality bases (Q < 20) and vector sequences, respectively. The turtle and alligator BESs can be found in the GenBank database under accession numbers CZ250707–CZ253982 (*A. mississipiensis*) and CZ253983–CZ257443 (*C. picta*) [38]. The emu sequences can be accessed via the NCBI Trace Archive website http://www.ncbi.nlm.nih.gov/Traces/trace.cgi.

Whole genome sequences for *Gallus gallus *were downloaded from the Washington University at St. Louis Genome Sequencing Center web site http://genome.wustl.edu/. The chicken assembly that was used here is version 2.1 from October 20, 2005 [5].

### BLAST analyses

For each species, a database containing its BESs was created. The BESs in a given database were compared to each other to detect any redundancy in sequence. Next, the three BES databases were compared to the chicken genome sequence using two different BLAST algorithms: BLASTN and TBLASTX. As a start, an E value cutoff of 10^-5 ^was selected for each BLAST analysis, without any alignment length criteria. The best matches in the chicken genome (up to 1000 for each blasted BAC end) were stored for each BES. BESs with no similarity to known repetitive elements (see "Repeated elements in the BESs" section) were annotated based on their most significant BLAST hit against the chicken genome (E value ≤ 10^-20^). For each BES, we designated as a "single BLAST hit" (SBH) the BLAST hit with the best e-value.

### MySQL database

All BLAST results were stored in relational MySQL http://www.mysql.com databases (one for each species). The structure of the MySQL databases and the relationships between the tables in them are presented in the Supplementary Material (Figure S1 in Additional File 1). Custom python scripts were used to create a toolbox that facilitated comparisons within and between the sequences databases, BLAST results, and statistical analyses (see below).

### Definition of a paired blast hit

We used the designation "paired BLAST hit" (PBH) to describe an instance when both BESs from a particular BAC exhibited significant alignment to the same chicken chromosome in the same orientation regardless of the distance between hits. Additionally, the qualifier "high quality" (hqPBH) was used to describe a PBH separated by ≤ 200 kb. Those PBHs that are not high quality were designated as "low quality" (lqPBH). For all the BESs from a species, we stored the alignment position(s) on the chicken genome assembly, the length of the alignment in nucleotides, the distance along the chicken alignment between both ends in the case of a PBH, and the E-values associated with alignments.

### Repeated elements in BESs

Visual analysis of the distribution of the number of paired hit sites per BAC clone and the distance between mapped hits in the chicken genome (see Results) suggested three classes of blast hits for each species: BAC clones yielding a large number of paired hits with a large genomic distance in the chicken (> 200 kb; lqPBH1); those yielding a small number of hits (1–4 in alligator and turtle and 1–7 in emu) and a large chicken genomic distance between them (lqPBH2); and finally those yielding a small number of hits and occurring < 200 kb apart in the chicken genome (hqPBHs).

RepeatMasker [43] was used on sequences for each of the three classes for each species in order to identify the content for repeat elements in the BESs. RepeatMasker accessed a database of consensus sequences of repeat elements for various mammalian species and chicken (Repbase version 20061006). The repeat content of the BAC clone sequences was assessed using default parameters without specifying the particular species queried or selecting human and/or chicken as the reference species. This protocol will likely miss many reptile-specific repeats since there are not many annotated reptile sequences in the databases, but it is a first step.

### Fingerprinting of the BAC clones

For each species, BAC clones that resulted in hqPBHs were selected for fingerprinting [44]. Thirty-four alligator clones and 24 turtle hqPBH clones were fingerprinted at the Washington University at St. Louis Genome Sequencing Center. As the number of hqPBHs for the emu was large, 50 BAC clones corresponding to hqPBHs from a single 384-well plate were selected for fingerprinting. Using the statistics software JMP version 7.0 from SAS http://www.jmp.com, the length of the BAC clone inserts and the average length of the corresponding paired hits on the chicken genome were compared for each species. The 95% confidence plot for each linear regression was used to assess statistical confidence.

### Gene content and statistics of BESs

The diversity of known genes in each hqPBH on the chicken genome was determined using the UCSC Genome Browser http://genome.ucsc.edu. The results are available as a spreadsheet in the Supplementary Material (Additional File 2). Using JMP, we performed statistical analyses (*χ*^2^, Van de Waerden's, Wald's and Student's *t*-tests) on means and distributions to test various hypotheses (see below).

## Results

### Blast hits for clones with one characterized sequence

4,095 of the 11,967 BESs from alligator, turtle and emu (34.2%) exhibited significant hits to the chicken genome using BLAST. Of the 3,218 sequences from alligator, 725 (22.5%) produced a total of 517,036 BLASTN hits. 773 of 3,461 turtle sequences (22.3%) generated 620,179 BLAST hits. 2,597 of 5,288 emu sequences (49.1%) generated a total of 972,993 significant hits. The vast majority (94%, 95% and 82% for alligator, turtle and emu respectively) of BAC clones with any significant E-values had a single characterized sequence, i.e. only one of the two BESs for a clone had a significant BLAST hit. The distributions of the number of BLAST hits per BES to the chicken genome for each species are presented in the Table 1. For the alligator, turtle and emu sequence sets, each BES hit on average 713, 802 and 375 sites in the chicken genome, respectively, although there was wide variation in the number of hits per BES. The most common result was to hit single sites in the chicken genome (Table 1). By contrast, between 4.5% (emu) and 6.3% (alligator) of clones with only one successfully BLASTed sequence matched the chicken genome at greater than 100 sites (Table 1). The emu had a larger number of clones with a small number (1–5) of hits, and a smaller number of clones with a large number of hits, than did alligator and turtle (Table 1). The distribution of the length of the blast hits is quite similar in the three query species (average hit length 38 ± 12 bp, 35 ± 21 bp, and 39 ± 14 bp, respectively), with a similar range in alligator and turtle (24 – 908 and 968 bp, respectively) and a somewhat shorter maximum in emu (24 – 680 bp; Supplementary Figure S1 in Additional File 1).

**Table 1:** Number of hits in chicken genome for clones with one significant end-sequence.

	Number of hits in chicken genome for clones with one significant end-sequence									
	
	1	2	3	4	5	5 < x < 20	20 ≤ x < 100	100 ≤ 1000	1000 < 10,000	> 10,000
	
Alligator	430 (13.4)	131 (4.1)	57 (1.8)	31 (1.0)	21 (0.7)	98 (3.0)	114 (3.5)	113 (3.5)	56 (1.7)	36 (1.1)
Turtle	479 (13.8)	171 (4.9)	85 (2.5)	36 (1.0)	29 (0.8)	127 (3.7)	69 (2.0)	70 (2.0)	64 (1.8)	41 (1.2)
Emu	1565 (29.6)	677 (12.8)	155 (2.9)	80 (1.5)	43 (0.8)	135 (2.6)	100 (1.9)	115 (2.2)	69 (1.3)	53 (1.0)

**Figure 1:**
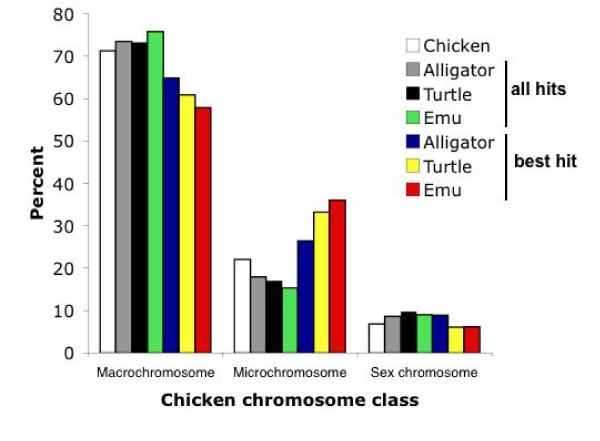
**Distribution of BLAST hits per BAC-end sequence for different chicken chromosomal classes**.

**Figure 2:**
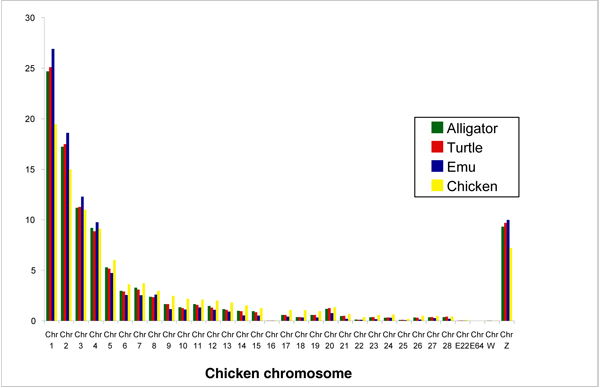
**Distribution of BLAST hits among chicken chromosomes**.

The diploid chicken genome is divided into 10 larger autosomes (macrochromosomes), W and Z sex chromosomes, and 66 microchromosomes. Various studies have revealed different evolutionary dynamics for these different types of chromosomes [5,45,46], prompting us to divide our analyses along those same lines. The number of hits on the chicken genome was therefore separated by macro-, micro- and sex chromosomes. In all three species, there is a broad correlation between the total number of hits for each chicken chromosomal class and the fraction of the chicken genome taken up by that chromosomal class (Figure 1; Supplementary Table S1 in Additional File 1). However, the observed number of hits on chicken macro-, micro- and sex chromosomes was nonetheless significantly different than their expected frequencies given the fraction of the genome taken up by these chromosomal classes (all tests df = 2; Alligator *χ*^2 ^= 6827; Turtle *χ*^2 ^= 15080; Emu *χ*^2 ^= 28717; all P < 0.00001). This difference also holds when considering only the chromosomal class occupied by the SBHs (alligator *χ*^2 ^= 22; turtle *χ*^2 ^= 83; emu *χ*^2 ^= 336; all P < 0.001). We then examined the distribution of hits for each individual chicken chromosome. For clones with a just a single successfully BLASTed BES, this broad correlation of hits and chromosome size extends to individual chicken chromosomes, whether considering all hits per sequence or only the best hit per sequence (SBH) (Figure 2; Supplementary Table S2 in Additional File 1). Nonetheless, for both situations, the distribution of total hits among chromosomes was significantly different from the distribution predicted by chicken chromosome size for all species (all tests df = 31; all hits: alligator *χ*^2 ^= 65113; turtle *χ*^2 ^= 90374; emu *χ*^2 ^= 360017; all P < 0.00001; single hits: alligator *χ*^2 ^= 83.9; turtle *χ*^2 ^= 121.7; emu *χ*^2 ^= 106.3; all P < 0.0001). The number of hits per chromosome was either significantly greater or less than expected for all chromosomes except chicken 30 (Wald's test; Supplementary Table S3 in Additional File 1). Even chicken chromosome 16, which comprises less than half a percent of the chicken genome, is represented by some hits from all three species, although fewer than expected (Figure 2; Supplementary Table S2 in Additional File 1). Considering all hits, the general pattern is that chicken macrochromosomes 1–4 (1–3 for turtle hits) and the Z chromosome are overrepresented by hits whereas all other chromosomes are underrepresented. Considering only the SBHs, alligator hits had a single overrepresented chicken chromosome (chr 1) and seven underrepresented; turtle showed four overrepresented (including chicken chr 1) and three underrepresented; and emu showed four significantly overrepresented chicken chromosomes and two underrepresented, including the Z.

The percent sequence identities of BLAST hits to the chicken genome in each chromosomal class were generally very high and similar among species, falling between 97.7 – 98.6%. In all species the distribution of identities showed a peak at the highest identity (> 99%) and a long tail down to 80–82%, depending on the species (Supplementary Figure S2 in Additional File 1). Nonetheless, *t*-tests show that the average percent identity among chicken macro-, micro- and sex chromosomes within each species showed significant variation (with the exception of the emu micro- and sex chromosomes); for alligator and turtle the identity for hits on the chicken sex chromosomes was significantly lower than for autosomes, whereas for emu hits on both micro- and sex chromosomes were lower than for macrochromosomes. For all chromosome classes the emu hits showed significantly higher sequence identity.

### Paired blast hits (PBHs)

We next examined for PBHs (those in the correct orientation on the same chicken chromosome, regardless of the distance between them on the chicken genome). By these criteria, approximately 3% of the alligator and turtle clones had at least one paired hit, whereas over 18% of the emu clones had paired blast hits on the chicken genome (Supplementary Table S4 in Additional File 1). The large number of hits for some clones with PHs could be explained by a large number of highly redundant hits (Table 2). As with clones with a single successfully BLASTed sequence, the distributions of the number of PBHs show that the vast majority of the BAC clones had very few paired hits, and the most common result was to have a single PBH (Table 2). The average length of BLAST hits from clones with PBHs was significantly greater than the length for hits from clones with a single successfully BLASTed end sequence (Supplementary Table S4 in Additional File 1).

**Table 2:** Number of hits in chicken genome for clones with PBHs.

	Number of hits in chicken genome for clones with two significant end-sequences									
	
	1	2	3	4	5	5 < x < 20	20 ≤ x < 100	100 ≤ 1000	1000 < 10,000	> 10,000
	
Alligator	27 (0.8)	9 (0.3)	7 (0.2)	2 (0.1)	1 (<0.1)	6 (0.2)	3 (0.1)	8 (0.2)	4 (0.1)	0 (0)
Turtle	29 (0.8)	9 (0.3)	0 (0)	0 (0)	1 (<0.1)	8 (0.2)	4 (0.1)	9 (0.3)	2 (0.1)	0 (0)
Emu	253 (4.8)	169 (3.2)	25 (0.5)	41 (0.8)	5 (0.1)	20 (0.4)	13 (0.2)	19 (0.4)	4 (0.1)	2 (0.0)

**Figure 3:**
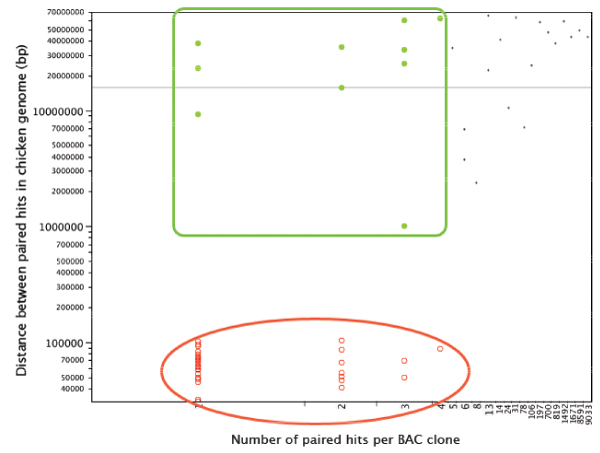
**Plot of the number of hits in the chicken genome per paired sequence from alligator for each BAC clone versus (x-axis) versus the distance between hits (y-axis)**. Each dot represents one BAC clone. For each BAC clone with a paired hit, the average lengths of the mapped hits on the chicken genome were computed. The y-axis scale is logarithmic. The plot is divided into three groups: clones with a large number of hits and a high intermarker distance for paired hits (black dots; lqPBH1); those with a small number of hits and a high intermarker distance for paired hits (green dots framed by a green square; lqPBH2); and finally those with a small number of hits and a small intermarker distance for paired hits ('high quality' paired hits, hqPBHs; red dots with encircling oval).

**Figure 4:**
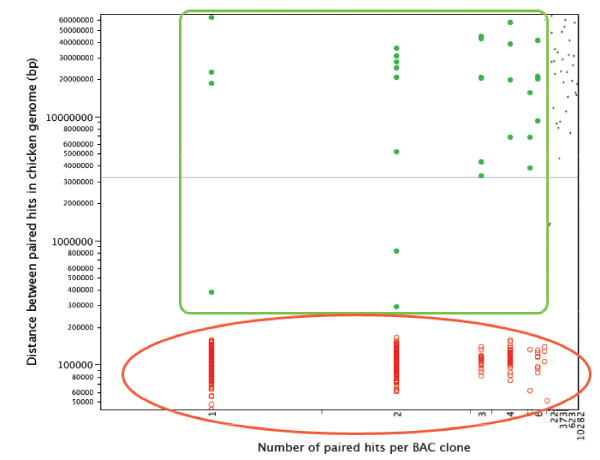
**Plot of the number of hits in the chicken genome per paired sequence from Turtle for each BAC clone versus (x-axis) versus the distance between hits (y-axis)**. Each dot represents a BAC clone. Details as in Figure 3.

**Figure 5:**
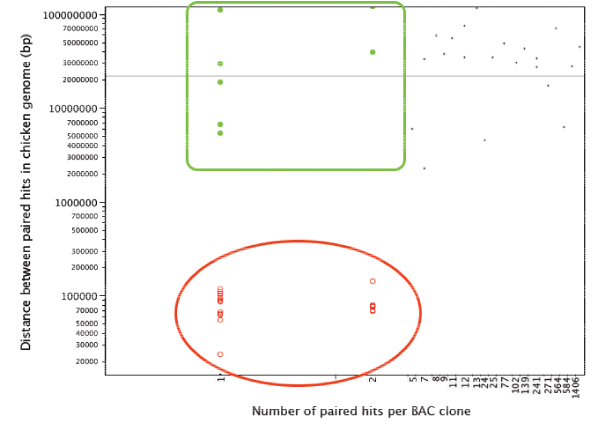
**Plot of the number of hits in the chicken genome per paired sequence from Emu for each BAC clone versus (x-axis) versus the distance between hits (y-axis)**. Each dot represents a BAC clone. Details as in Figure 3.

For all three species the average distances between PBHs on the chicken genome ranged from 10 kb to more than 100 Mb (Figures 3, 4, 5). However, for each analysis, the plots could be divided into three distinct groups: lqPBH1 and 2, and hqPBH (see Methods for definitions).

We focused on the hqPBHs. We found that for all three species, hqPBHs nearly always have a small number of hits in the chicken genome. (In many cases the number of sites of hqPBHs in the chicken genome is even smaller than we have indicated in Figures 3, 4, 5 because the genomic coordinates of different pairs are in all cases virtually identical, within 100 bp of each other and usually much less [see Supplementary Material – Additional File 2]. Therefore each BAC clone with hqPBHs could in fact be represented by only one hit.) The number of clones with hqPBHs was a very small percentage of the total number of clones for both alligator and turtle, but for emu this percentage was moderate (Supplementary Tables S2 and S4 in Additional File 1). In the emu a larger number of PBHs occurring less then 200 kb apart in the chicken genome mapped to multiple sites in the chicken genome (Table 2). On average, hqPBHs from emu were significantly farther apart in the chicken genome (mean 108,079 ± 19,788 bp) than in the alligator or turtle (means 65,306 ± 19,324 bp and 86,487 ± 23,934 bp, respectively; Supplementary Figure S4 in Additional File 1).

**Figure 6:**
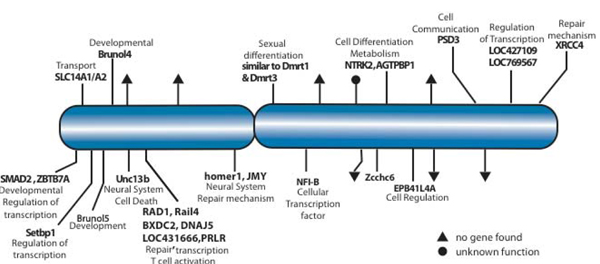
**Correlation between the intermarker distances in the chicken genome (y-axis) and in Alligator (A), Turtle (B) and Emu (C) (x-axis)**. Each tick represents a single emu BAC clone. The gene names inferred to occur on that BAC clone and their GO functions are listed.

The distribution among chicken chromosomes of hqPBHs showed a weaker relationship to chromosome size for all species, presumably due to smaller sample size (Supplementary Figure S3 and Supplementary Table S2 in Additional File 1). The number of hqPBHs for turtle was significantly overrepresented on chicken chromosomes 1 and 2 and for emu hits on chicken chromosomes 12, 20 and (marginally) 26; emu hqPBHs on chicken Z were marginally underrepresented (Supplementary Table S3 in Additional File 1). The hqPBHs from alligator are mainly on macrochromosomes and none occur on chicken sex chromosomes. The turtle hqPBHs are equally represented on macro- and microchromosomes but again are not present on chicken sex chromosomes. By contrast, the emu hqPBHs have orthologous sites on 25 chromosomes of all three chromosomal classes. The number of emu hqPBHs on chicken chromosomes was tightly correlated with the size of the chicken chromosome (R^2 ^= 0.965, P < 0.0001; Supplementary Figure S5 in Additional File 1). The locations of emu hqPBHs emu on the chicken Z chromosome are shown in Figure 6 (see below).

### Correlations among interspecific genomic distances

**Figure 7:**
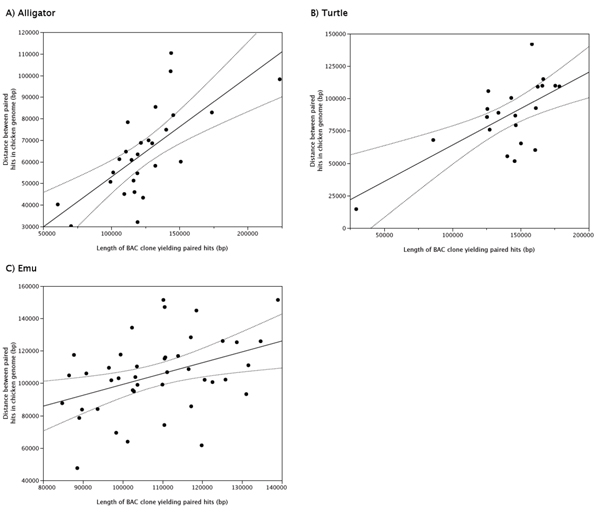
**Schematic of the chicken Z chromosome and the location of emu BAC clones with high quality paired hits**. Each dot represents the average length of the high quality paired mapped hits for one BAC clone. The linear regression and the confidence curve fits are drawn in thick and dotted lines, respectively. The estimated parameters and P-values for the regression for each species are: Alligator: slope, 0.46; intercept 6570; R^2^ = 0.48, P < 0.0001. Turtle: slope, 0.56; intercept 7680; R^2^ = 0.45, P < 0.0001. Emu: slope, 0.67; intercept 32240; R^2 ^= 0.16, P < 0.09.

To quantify the relationship between genomic distances between markers in the chicken genome and in the genomes of alligator, turtle and emu, the insert sizes of BAC clones with an hqPBH were determined via fingerprinting. All hqPBH alligator and turtle BAC clones were fingerprinted (of which 27 and 22 were successful) and among the 479 emu BAC clones with hqPBH(s) we chose 50 that appeared to span a wide range of distances in the chicken genome (~40 – 160 kb). The results of eight emu clones were disregarded because estimated insert sizes were unrealistically large (~300 kb). Figure 7A-C shows the correlation between the lengths of the hqPBHs mapped on the chicken genome and the estimated insert sizes of the corresponding BAC clones. The regressions of the alligator and turtle BAC clones are significant with slopes close to 0.50, remarkably close to the ratio of the genome sizes compared to chicken (0.50 for alligator and 0.48 for turtle). The emu linear regression had a slope of 0.67, again close to that predicted by the ratio of genome sizes (0.77), but was not significant.

### Analysis of repetitive elements

We studied the genomic content of the BAC end data set, as well as any consistent differences between classes of BAC clones, using RepeatMasker. For each species, three groups of clones were screened (Table 3): the complete BAC-end data set ('total'), clones with hqPBH(s), and the remaining sequences involved in paired hits (lqPBHs1 and 2). The alligator and turtle data sets revealed significant differences in repeat element content in the three groups of data. The sequences involved in hqPBHs have significantly fewer repeat elements (5× or 2× less for alligator or turtle, respectively) than their respective total data sets or lqPBHs. This is especially the case for the non-long-terminal repeat retrotransposons of the chicken Repeat 1 (CR1) class. The sequences in lqPBHs of the alligator and turtle have 22 and 10 times more CR1 elements respectively than the hqPBH sequences. By contrast, the hqPBH sequences of the emu clones have the same repeat element content as the total data set. The emu lqPBH sequences have a slightly higher number of repeat elements.

**Table 3:** Repeated element content of different fractions of BAC-end sequences from American Alligator, Painted Turtle and Emu.

	Alligator			Turtle			Emu		
	total	high quality hits	low quality hits	total	high quality hits	low quality hits	total	high quality hits	low quality hits

Data set size (Mb)	2.5	0.056	0.047	2.4	0.051	0.049	3.5	0.52	0.073

Percent repeat element content									
SINE (MIRs)	0.72	0.42	1.44	1.78	2.76	1.96	0	0	0
LINEs	7.64	1.34	27.87	4.61	0.71	15.15	1.25	1.30	2.16
L2/CR1/Rex	7.62	1.25	27.87	4.43	0.16	15.01	1.25	1.30	2.16
LTR	0.22	0	0	0.09	0	0	0.99	0.92	1.13
DNA transposons	0.1	0	0.1	0.20	0	12.91	0	0	0
microsatellites	0.33	0.08	1.5	0.35	0.31	0.12	0.38	0.07	1.42
Total interpersed repeats	8.68	1.76	29.45	6.68	3.47	17.11	2.24	2.22	3.29
Small RNA	0.22	0	2.30	0.35	0	12.91	0	0	0

### Gene content

To keep our analysis of gene content focused on those results of highest priority for comparative mapping purposes, using the UCSC Genome Browser we inferred gene content only for the 537 BAC clones with hqPBHs across species. We mapped the two end-sequences to the chicken genome then queried the Genome Browser as to which gene occurred in the chicken genome between the two landmarks. Thus there is a single inferred gene set of each BAC clone. The complete results are included in Supplementary Material (Additional File 2). Gene predictions included many of interest to the biology of birds and reptiles, including 25 clones mapping to all portions of the chicken Z chromosome. These mapped genes provide useful predictions for the gene content of the original BAC clones. For example, emu BAC clone B10 in plate 280 is predicted to contain sequences similar to doublesex and mab-3 related transcription factors DMRT1 and DMRS3. Intriguingly, this emu sequence mapped with high confidence to the chicken Z chromosome as predicted by the genome sequence and genetic and physical maps [47]. DMRS1 is the only gene known to distinguish the large and nearly identical Z and W chromosomes of emu [48]. This clone may therefore provide an insight into the evolutionary history of this gene family potentially involved in sex determination and other critical functions.

## Discussion

We have mapped *in silico *11,967 BESs from three Reptilia, including a basal bird: American alligator, painted turtle and an emu. These three species are phylogenetically well placed to better understand the origin of the chicken genome and to put more detail on evolutionary events in the Reptilia, the amniote group that is the sister group of mammals. Recently hybridization methods, such as FISH and array CGH [49], as well as *in silico *mapping of BAC sequences [50], have been applied to the comparison of two relatively closely related birds, chicken and turkey. These studies, as well as previous chromosomal work in birds, have found a relatively conservative pattern of chromosomal inversions, due both to the recency of divergence of these species and to the overall conservative mode of chromosome evolution in birds, and possibly the Reptilia in general [46,51]. This conservatism extends to cross-hybridization studies between birds and non-avian reptiles such as turtles [6,9]. FISH studies tend to detect broad-scale synteny and are little affected by intrachromosomal micro- or even BAC-scale changes in synteny. By contrast, *in silico *mapping of BESs between highly diverged species such as we have examined here results in higher stringency tests for conservation of synteny, and on a scale complementary to that provided by techniques such as FISH. Despite the high temporal and sequence divergence of comparisons in our study, the relative frequency of significant BLAST hits for both single sequence BACs and PBHs is consistent with the relative divergence times of the alligator, turtle and emu from chicken.

As expected, the emu sequences had the highest frequency of significant BLAST hits (~50%) to the chicken genome. The divergence time between the ratites and the Galliformes is around 120 My – about half the divergence time between chicken and alligator (220 My) and that of chicken and turtle (240 My) [11,52,53]. The similarities of the emu sequences with the chicken genome are confirmed by the small number of hits per BAC clone sequence and the fact that these hits are generally longer than those of the two non-avian reptiles. For example, 870 emu BAC-end sequences (34% of the BAC-end sequences with hits) have at least one hit longer than 200 bp. By contrast, the proportion of large hits is significantly smaller for the alligator and turtle sequences: only 16% and 15% of their respective BAC-end sequences have a blast hit longer than 200 bp.

The analyses of clones with PBHs revealed that their frequency in the emu was roughly five times greater than for alligator or turtle, as compared with roughly two times higher frequency of single hits. This difference is even higher (eight-fold greater) when considering only hqPBHs. Whereas 16% of the emu clones had hqPBH(s), the turtle and the alligator BAC clones have 1.3% and 2% such hits. These results confirm that the emu genome has retained a larger number of areas of microsyntenic regions since the divergence of modern birds as compared with the alligator and turtle. Griffin et al. [54] suggested that retention of microsynteny should be the rule in avian genomes. In an analogous study using PCR, Thomson et al. [39] recently showed that the fraction of BESs from the painted turtle library that could be reliably amplified from other turtles decayed linearly with divergence time from the painted turtle time at a rate of -0.25 to -0.32% amplifiability per million years; the fraction of painted turtle loci amplified from target species fell from over 80% among recently diverged species to less than 20% in some cases for species diverged more than 200 MYA.

The analysis of repeated elements in our BAC end sequences is also consistent with what we know about genome structure in reptiles. In the alligator and the turtle, the repeat element content is relatively high compared to birds [40], thus we expected that alligator and turtle BESs would contain more repeat elements than the emu sequences. The chicken genome is approximately 12% repeat elements, which are widely dispersed throughout the genome and dominated by the CR1 family of LINEs [5,55]. As a result, BACs in which both sequences contain repeated elements often had very high hit numbers, regardless of sequence length or insert size. This probably underlies the association between high hit number and very large inferred regions of the chicken genome delimited by paired markers (Figures 3, 4, 5). The difference in repeat element content between hqPBHs and lqPBH groups 1 and 2 was pronounced in alligator and the turtle, whereas the emu sequences appears to have a relatively low number of repeat elements (~2.5–3%), even as compared to other birds [49]. Whether or not this low repeat content extends to the genome as a whole is an unanswered question, but we have noticed very low repeat element contents in fully sequenced BACs in the online data bases (A. Shedlock, D. Janes, S. Edwards, unpubl.). This pattern contrasts with the significantly larger genome size of emus relative to chicken, which is routinely assumed to be due to proliferation of repeated elements. The effect of this low repeat element content in emu is to minimize the number of spurious, non-unique hits to the chicken genome, which might explain the high proportion of hqPBHs.

hqPBHs of the emu are present in nearly all chromosomes. The emu was the only species to have any hqPBHs on the chicken Z chromosome. Comparisons of physical and genetic maps among various bird lineages suggest that this chromosome is highly conserved in its gene order [56-59]. The emu Z and W chromosomes are similar in size and as large as some chicken macro chromosomes; chromosome painting revealed that the emu Z and W and chicken Z chromosomes are broadly homologous across their entire length [60]. In addition the psuedoautosomal region of the emu Z chromosome exhibits levels of nucleotide diversity and recombination that are similar to emu autosomes [61]. There was one emu clone whose best single hit was on the chicken W (Supplementary Table S2 in Additional File 1); whether this represents a bona fide homology remains to be seen. That the coverage of the chicken W is still poor in draft chicken genome release 2.1 and that the W is very small in chickens may also explain this result. Regardless, this study has identified numerous clones that can be characterized to better understand the emu sex chromosomes and autosomes. It is unclear whether the lack of alligator or turtle hqPBHs on the chicken Z is due to high levels of sequence divergence, a lack of sex chromosomes in these species (both have temperature dependent sex determination) or insufficient coverage in our survey. Turtle chromosome 5 was found to be completely homologous to the chicken Z chromosome [6], and so it may be that the anonymous sequences available through BAC ends are too diverged from chicken for *in silico *mapping. Certainly there are many alligator and turtle BAC clones whose SBH is on the Z chromosome, and these warrant further investigation.

The correlation of the distance between genomic markers of different species and chicken was high for alligator and turtle, but less strong for the emu, even though the emu comparison had a higher number of intervals for comparison. In each case the slope of the fit was close to the ratio of genome sizes between query and target species. The weak relationship in emu could be a result of the more similar genome sizes between the emu and chicken (1.63 Gb vs. 1.25 Gb, respectively), a difference of only 30% of the chicken genome size. By contrast, the alligator and turtle have genome sizes roughly double that of chicken. Nonetheless the high correlation in all species suggests that there was a contribution to genome size reduction in the avian lineage of many small deletions on the size scale of BAC clones. The small genomes of birds are thought to have arisen deep within the therapod dinosaurian lineage from which birds evolved, and yet the deletion of retroelements from amniote ancestors has been estimated to comprise only 15–20% of this reduction in genome size [40]. Our analysis of hqPBHs in particular suggests that single- or low-copy regions of the genome have also experienced reductions in size. In some lineages of birds there appears to be a bias toward deletions comprising a few base pairs, and this could also have contributed to maintenance of small genomes in birds.

## Conclusion

BAC libraries from non-model species are powerful resources for studying genome evolution in a comparative context. Our results suggest a large number of BAC-scale chromosomal rearrangements and deletions in chicken relative to alligator and turtle, and fewer such rearrangements compared to emu. The study also suggests a substantial level of divergence at the level of sequences between these species as detected in BLAST analyses. The analysis shows many small deletions dispersed throughout the ancestral amniotes and reptile genomes contributed to the overall reduction in genome size in birds. Our study has also flagged hundreds of easily locatable BAC clones from two reptiles and a basal bird that are predicted to contain specific regions of the chicken genome and which can now be mined for specific genes and verified as to chromosomal location via molecular methods. Ultimately, sequencing of many of these BAC will provide an even clearer picture of the sequence of events leading to the streamlined genomes of birds at the nucleotide level as well as the details of evolution of many gene regions of interest to geneticists and developmental biologists.

## List of abbreviations used

BAC: bacterial artificial chromosome; BES: BAC end sequence; SBH: single BLAST hit; PBH: paired BLAST hit; hqPBH: high quality paired BLAST hit; lqPBH: low quality paired BLAST hit.

## Competing interests

The authors declare that they have no competing interests.

## Authors' contributions

The project was conceived by SVE, and CC and SVE designed the research protocol. CC developed the bioinformatics pipeline, developed the database, and produced the results. CC and SVE wrote the paper.

## Supplementary Material

Additional file 1 Additional statistics of BLAST analyses on chicken chromosomes, including number of hits per chromosome, Wald's tests for observed and expected number of hits per chromosome, percent identities and lengths of hits. Click here for file

Additional file 2 Spreadsheet listing inferred gene contents of alligator, turtle and emu BACs with high quality paired hits to chicken chromosomes Click here for file

## References

[B1] Waterston RH Lindblad-Toh K Birney E Rogers J Abril JF Agarwal P Agarwala R Ainscough R Alexandersson M An P Initial sequencing and comparative analysis of the mouse genome Nature 2002 420 520 562 12466850 10.1038/nature01262

[B2] Zhao S Shatsman S Ayodeji B Geer K Tsegaye G Krol M Gebregeorgis E Shvartsbeyn A Russell D Overton L Mouse BAC ends quality assessment and sequence analyses Genome Res 2001 11 1736 1745 11591651 10.1101/gr.179201 PMC311142

[B3] Newman TL Tuzun E Morrison VA Hayden KE Ventura M McGrath SD Rocchi M Eichler EE A genome-wide survey of structural variation between human and chimpanzee Genome Res 2005 15 1344 1356 16169929 10.1101/gr.4338005 PMC1240076

[B4] Consortium ICGS Sequence and comparative analysis of the chicken genome provide unique perspectives on vertebrate evolution Nature 2004 432 695 716 15592404 10.1038/nature03154

[B5] Burt DW Chicken genome: current status and future opportunities Genome Res 2005 15 1692 1698 16339367 10.1101/gr.4141805

[B6] Graves JAM Sex from W to Z: Evolution of vertebrate sex chromosomes and sex determining genes Journal of Experimental Zoology 2001 290 449 462 11555852 10.1002/jez.1088

[B7] Kawai A Different origins of bird and reptile sex chromosomes inferred from comparative mapping of chicken Z-linked genes Cytogenetic and Genome Research 2007 117 92 102 10.1159/000103169 17675849

[B8] Yamada K Nishida-Umehara C Matsuda Y Molecular and cytogenetic characterization of site-specific repetitive DNA sequences in the Chinese soft-shelled turtle (Pelodiscus sinensis, Trionychidae) Chromosome Res 2005 13 33 46 15791410 10.1007/s10577-005-2351-0

[B9] Matsuda Y Nishida-Umehara C Tarui H Kuroiwa A Yamada K Isobe T Ando J Fujiwara A Hirao Y Nishimura O Highly conserved linkage homology between birds and turtles: bird and turtle chromosomes are precise counterparts of each other Chromosome Res 2005 13 601 615 16170625 10.1007/s10577-005-0986-5

[B10] Cao Y Sorenson MD Kumazawa Y Mindell DP Hasegawa M Phylogenetic position of turtles among amniotes: evidence from mitochondrial and nuclear genes Gene 2000 259 139 148 11163971 10.1016/s0378-1119(00)00425-x

[B11] Hedges SB Poling LL A molecular phylogeny of reptiles Science 1999 283 998 1001 9974396 10.1126/science.283.5404.998

[B12] Iwabe N Hara Y Kumazawa Y Shibamoto K Saito Y Miyata T Katoh K Sister group relationship of turtles to the bird-crocodilian clade revealed by nuclear DNA-coded proteins Mol Biol Evol 2005 22 810 813 15625185 10.1093/molbev/msi075

[B13] Rest JS Ast JC Austin CC Waddell PJ Tibbetts EA Hay JM Mindell DP Molecular systematics of primary reptilian lineages and the tuatara mitochondrial genome Mol Phylogenet Evol 2003 29 289 297 13678684 10.1016/s1055-7903(03)00108-8

[B14] Zardoya R Meyer A Complete mitochondrial genome suggests diapsid affinities of turtles Proc Natl Acad Sci USA 1998 95 14226 14231 9826682 10.1073/pnas.95.24.14226 PMC24355

[B15] Meyer A Zardoya R Recent advances in the (molecular) phylogeny of vertebrates Annu Rev Ecol Evol Syst 2003 34 311 318

[B16] Bonneaud C Burnside J Edwards SV High-speed developments in avian genomics Bioscience 2008 58 587 595

[B17] Hackett SJ Kimball RT Reddy S Bowie RC Braun EL Braun MJ Chojnowski JL Cox WA Han KL Harshman J A phylogenomic study of birds reveals their evolutionary history Science 2008 320 1763 1768 18583609 10.1126/science.1157704

[B18] Nakamura D Tiersch TR Douglass M Chandler RW Rapid identification of sex in birds by flow cytometry Cytogenetics and Cell Genetics 1990 53 201 205 10.1159/000132930 2209087

[B19] Roots EH Baker RJ Distribution and characterization of microsatellites in the emu (Dromaius novaehollandiae) genome J Hered 2002 93 100 106 12140269 10.1093/jhered/93.2.100

[B20] Tiersch TR Wachtel SS On the evolution of genome size of birds Journal of Heredity 1991 82 363 368 1940280 10.1093/oxfordjournals.jhered.a111105

[B21] Nishida-Umehara C Tsuda Y Ishijima J Ando J Fujiwara A Matsuda Y Griffin DK The molecular basis of chromosome orthologies and sex chromosomal differentiation in palaeognathous birds Chromosome Res 2007 15 721 734 17605112 10.1007/s10577-007-1157-7

[B22] Mirsky AE Ris H The desoxyribonucleic acid content of animal cells and its evolutionary significance Journal of General Physiology 1951 451 462 14824511 10.1085/jgp.34.4.451 PMC2147229

[B23] Vinogradov AE Genome size and GC-percent in vertebrates as determined by flow cytometry: the triangular relationship Cytometry 1998 31 100 109 9482279 10.1002/(sici)1097-0320(19980201)31:2<100::aid-cyto5>3.0.co;2-q

[B24] Bull JJ Sex determination in reptiles Quart Rev Biol 1980 1 21

[B25] Valleley EM Harrison CJ Cook Y Ferguson MW Sharpe PT The karyotype of Alligator mississippiensis, and chromosomal mapping of the ZFY/X homologue, Zfc Chromosoma 1994 103 502 507 7720416 10.1007/BF00337388

[B26] Bickham JW Baker RJ Chromosome Homology and Evolution of Emydid Turtles Chromosoma 1976 54 201 219 1248339 10.1007/BF00293451

[B27] Zhao S Stodolsky M Library construction, physical mapping and sequencing Bacterial artificial chromosomes 2004 1 1 Humana Press

[B28] Zhao S Stodolsky M Functional studies Bacterial artificial chromosomes 2004 2 2 Totowa, NJ: Humana Press

[B29] Volik S Zhao S Chin K Brebner JH Herndon DR Tao Q Kowbel D Huang G Lapuk A Kuo WL End-sequence profiling: Sequence-based analysis ofaberrant genomes Proc Natl Acad Sci USA 2003 100 7696 7701 12788976 10.1073/pnas.1232418100 PMC164650

[B30] Liu G Zhao S Bailey JA Sahinalp SC Alkan C Tuzun E Green ED Eichler EE Analysis of primate genomic variation reveals a repeat-driven expansion of the human genome Genome Res 2003 13 358 368 12618366 10.1101/gr.923303 PMC430288

[B31] Lai CW Yu Q Hou S Skelton RL Jones MR Lewis KL Murray J Eustice M Guan P Agbayani R Analysis of papaya BAC end sequences reveals first insights into the organization of a fruit tree genome Mol Genet Genomics 2006 276 1 12 16703363 10.1007/s00438-006-0122-z

[B32] Hong CP Lee SJ Park JY Plaha P Park YS Lee YK Choi JE Kim KY Lee JH Lee J Construction of a BAC library of Korean ginseng and initial analysis of BAC-end sequences Mol Genet Genomics 2004 271 709 716 15197578 10.1007/s00438-004-1021-9

[B33] Wang CM Lo LC Feng F Gong P Li J Zhu ZY Lin G Yue GH Construction of a BAC library and mapping BAC clones to the linkage map of Barramundi, Lates calcarifer BMC Genomics 2008 9 139 18366732 10.1186/1471-2164-9-139 PMC2329641

[B34] Dalrymple BP Kirkness EF Nefedov M McWilliam S Ratnakumar A Barris W Zhao S Shetty J Maddox JF O'Grady M Using comparative genomics to reorder the human genome sequence into a virtual sheep genome Genome Biol 2007 8 R152 17663790 10.1186/gb-2007-8-7-r152 PMC2323240

[B35] Eichler EE DeJong PJ Biomedical applications and studies of molecular evolution: a proposal for a primate genomic library resource Genome Res 2002 12 673 678 11997334 10.1101/gr.250102

[B36] Locke DP Segraves R Carbone L Archidiacono N Albertson DG Pinkel D Eichler EE Large-scale variation among human and great ape genomes determined by array comparative genomic hybridization Genome Res 2003 13 347 357 12618365 10.1101/gr.1003303 PMC430292

[B37] Dawson DA Burke T Hansson B Pandhal J Hale MC Hinten GN Slate J A predicted microsatellite map of the passerine genome based on chicken-passerine sequence similarity Mol Ecol 2006 15 1299 1320 16626455 10.1111/j.1365-294X.2006.02803.x

[B38] Shedlock AM Phylogenomic investigation of CR1 LINE diversity in reptiles Syst Biol 2006 55 902 911 17345672 10.1080/10635150601091924

[B39] Thomson RC Shedlock AM Edwards SV Shaffer HB Developing markers for multilocus phylogenetics in non-model organisms: a test case with turtles Molecular Phylogenetics and Evolution 2008 49 514 525 10.1016/j.ympev.2008.08.006 18761096

[B40] Shedlock AM Janes D Edwards SV Murphy W Amniote phylogenomics: Testing hypotheses with large-scale sequence data from reptiles Phylogenomics 2008 Totowa, N.J.: Humana Press 91 117 10.1007/978-1-59745-581-7_7 18629663

[B41] Ewing B Hillier L Wendl MC Green P Base-calling of automated sequencer traces using phred. I. Accuracy assessment Genome Res 1998 8 175 185 9521921 10.1101/gr.8.3.175

[B42] Altschul SF Gish W Miller W Myers EW Lipman DJ Basic local alignment search tool J Mol Biol 1990 215 403 410 2231712 10.1016/S0022-2836(05)80360-2

[B43] Smit AFA Hubley R Green P RepeatMasker Open-3.0 1996

[B44] Schein J Kucaba T Sekhon M Smailus D Waterston R Marra M Zhao S, Stodolsky M High-throughput BAC Fingerprinting Bacterial Artificial Chromosomes 2004 255 Humana Press 143 156 10.1385/1-59259-752-1:143 15020821

[B45] Axelsson E Webster MT Smith NG Burt DW Ellegren H Comparison of the chicken and turkey genomes reveals a higher rate of nucleotide divergence on microchromosomes than macrochromosomes Genome Res 2005 15 120 125 15590944 10.1101/gr.3021305 PMC540272

[B46] Burt DW Bruley C Dunn IC Jones CT Ramage A Law AS Morrice DR Paton IR Smith J Windsor D The dynamics of chromosome evolution in birds and mammals Nature 1999 402 411 413 10586880 10.1038/46555

[B47] Itoh Y Kampf K Arnold AP Comparison of the chicken and zebra finch Z chromosomes shows evolutionary rearrangements Chromosome Res 2006 14 805 815 17139532 10.1007/s10577-006-1082-1

[B48] Shetty S Kirby P Zarkower D Graves JA DMRS1 in a ratite bird: evidence for a role in sex determination and discovery of a putative regulatory element Cytogenet Genome Res 2002 99 245 251 12900571 10.1159/000071600

[B49] Griffin DK Robertson LB Tempest HG Vignal A Fillon V Crooijmans RP Groenen MA Deryusheva S Gaginskaya E Carre W Whole genome comparative studies between chicken and turkey and their implications for avian genome evolution BMC Genomics 2008 9 168 18410676 10.1186/1471-2164-9-168 PMC2375447

[B50] Reed KM Faile GM Kreuth SB Chaves LD Sullivan LM Association and in silico assignment of sequences from turkey BACs Anim Biotechnol 2008 19 80 83 18432398 10.1080/10495390701876209

[B51] Burt DW Origin and evolution of avian microchromosomes Cytogenetics and Genome Research 2002 96 97 112 10.1159/000063018 12438785

[B52] Edwards SV Jennings WB Shedlock AM Phylogenetics of modern birds in the era of genomics Proceedings of the Royal Society B-Biological Sciences 2005 272 979 992 10.1098/rspb.2004.3035 PMC1599873 16024355

[B53] Kumar S Hedges SB A molecular timescale for vertebrate evolution Nature 1998 392 917 920 9582070 10.1038/31927

[B54] Griffin DK Robertson LB Tempest HG Skinner BM The evolution of the avian genome as revealed by comparative molecular cytogenetics Cytogenet Genome Res 2007 117 64 77 17675846 10.1159/000103166

[B55] Wicker T Robertson JS Schulze SR Feltus FA Magrini V Morrison JA Mardis ER Wilson RK Peterson DG Paterson AH The repetitive landscape of the chicken genome Genome Res 2005 15 126 136 15256510 10.1101/gr.2438005 PMC540276

[B56] Akesson M Hansson B Hasselquist D Bensch S Linkage mapping of AFLP markers in a wild population of great reed warblers: importance of heterozygosity and number of genotyped individuals Mol Ecol 2007 16 2189 2202 17561884 10.1111/j.1365-294X.2007.03290.x

[B57] Backstrom N Karaiskou N Leder EH Gustafsson L Primmer CR Qvarnstrom A Ellegren H A gene-based genetic linkage map of the Collared Flycatcher (*Ficedula albicollis*) reveals extensive synteny and gene-rrder conservation during 100 Million years of avian evolution Genetics 2008 179 1479 1495 18562642 10.1534/genetics.108.088195 PMC2475748

[B58] Dawson DA Akesson M Burke T Pemberton JM Slate J Hansson B Gene order and recombination rate in homologous chromosome regions of the chicken and a passerine bird Mol Biol Evol 2007 24 1537 1552 17434902 10.1093/molbev/msm071

[B59] Stapley J Birkhead TR Burke T Slate J A linkage map of the zebra finch *Taeniopygia guttata *provides new insights into avian genome evolution Genetics 2008 179 651 667 18493078 10.1534/genetics.107.086264 PMC2390641

[B60] Shetty S Comparative painting reveals strong chromosome homology over 80 million years of bird evolution Chromosome Research 1999 7 289 295 10461874 10.1023/a:1009278914829

[B61] Janes DE Ezaz T Graves JAM Edwards SV Recombination and nucleotide diversity in the pseudoautosomal region of minimally differentiated sex chromosomes in the Emu, *Dromaius novaehollandiae* Journal of Heredity 2008 100 125 136 18775880 10.1093/jhered/esn065 PMC2734100

