# Supplementary material for: Genome evolution in Reptilia: in silico chicken mapping of 12,000 BAC-end sequences from two reptiles and a basal bird
Source: BMC Genomics. 2009 Jul 14;10(Suppl 2):S8. doi: 10.1186/1471-2164-10-S2-S8 (PMC2966332; doi:10.1186/1471-2164-10-S2-S8)
Supplement: Additional file 1 — Additional statistics of BLAST analyses on chicken chromosomes, including number of hits per chromosome, Wald's tests for observed and expected number of hits per chromosome, percent identities and lengths of hits. [file 1471-2164-10-S2-S8-S1.doc]

**Supplementary Material**

**for**

**“Genome evolution in Reptilia: *In silico* mapping to the chicken genome of 12,000 BAC-end sequences from two reptiles and a basal bird”**

**by**

Charles Chapus and Scott V. Edwards

I. Schematic diagram of MySQL data base for analysis of BAC-end sequences. [2](#__RefHeading___Toc80663209)

II. Tables [3](#__RefHeading___Toc80663210)

A. Supplementary Table 1. Number (percentage) of hits per chromosomal class for BAC-end sequences from the three query species. [3](#__RefHeading___Toc80663211)

B. Supplementary Table 2. Numbers of hits per chicken chromosome for single- and paired hit BLAST results. [4](#__RefHeading___Toc80663212)

C. Supplementary Table 3. Wald’s tests of the distribution of hits among chicken chromosomes for various subsets of the data. [5](#__RefHeading___Toc80663213)

D. Supplementary Table 4. Statistics of BAC clones with paired hits to the chicken genome. [6](#__RefHeading___Toc80663214)

III. Figures [7](#__RefHeading___Toc80663215)

A. Figure 1. Distribution of lengths of BLAST hits for all significant hits [7](#__RefHeading___Toc80663216)

B. Figure 2. Distribution of percent identities for all hits. [8](#__RefHeading___Toc80663217)

C. Figure 3. Distribution of high quality paired hits per chicken chromosome [9](#__RefHeading___Toc80663218)

D. Figure 4. Distribution of intermarker distances in the chicken genome for high quality paired hits for alligator, turtle and emu. [10](#__RefHeading___Toc80663219)

E. Correlation between the number of high quality paired hits for emu and size of the target chicken chromosome [11](#__RefHeading___Toc80663220)

F. Supplementary spreadsheet listing inferred gene contents of BACs with high quality paired hits [11](#__RefHeading___Toc80663221)

# Schematic diagram of MySQL database for analysis of BAC-end sequences.

# Tables

## Supplementary Table 1. Number (percentage) of hits per chromosomal class for BAC-end sequences from the three query species.

This table focuses on only those BAC clones with a single successfully BLASTed –end sequence.

|  | Macrochromosome | Microchromosome | Z chromosome |
| --- | --- | --- | --- |
|  | Percent of chicken genome | | |
| **Chicken** | (71.2) | (22.0) | (6.8) |
|  | Number (percent) of hits, considering all significant hits per clone | | |
| **Alligator** | 379,850  (73.5) | 92,607  (17.9) | 44,579  (8.6) |
| **Turtle** | 454,355  (73.3) | 105,591  (17.0) | 60,233  (9.7) |
|
| **Emu** | 737,317  (75.8) | 149,229  (15.3) | 86,447  (8.9) |
|  | Number (percent) of hits, considering only single best hit per clone | | |
| **Alligator** | 686  (64.8) | 279  (26.3) | 94  (8.9) |
| **Turtle** | 699  (60.8) | 381  (33.2) | 69  (6.0) |
| **Emu** | 1698  (57.9) | 1056  (36.0) | 180  (6.1) |

## Supplementary Table 2. Numbers of hits per chicken chromosome for single- and paired hit BLAST results.

|  |  | Number of BLAST hits | | | | | | | | |
| --- | --- | --- | --- | --- | --- | --- | --- | --- | --- | --- |
|  |  | All hits for clones with one significant sequence | | | Single best hit for clones with one significant sequence | | | Clones with high quality paired hits | | |
| Chicken chromosome | Percentage of chicken genome | Alligator | Turtle | Emu | Alligator | Turtle | Emu | Alligator | Turtle | Emu |
| Chr 1 | 19.5 | 287251 | 346137 | 764990 | 287 | 261 | 571 | 6 | 2 | 82 |
| Chr 2 | 15.0 | 200609 | 241137 | 528904 | 132 | 159 | 405 | 3 | 1 | 72 |
| Chr 3 | 11.0 | 130119 | 155424 | 349604 | 121 | 124 | 325 | 2 | 2 | 55 |
| Chr 4 | 9.1 | 107053 | 122346 | 277445 | 98 | 88 | 247 | 5 | 2 | 34 |
| Chr 5 | 6.0 | 61649 | 71167 | 134615 | 48 | 67 | 150 | 4 | 2 | 25 |
| Chr 6 | 3.6 | 34527 | 40183 | 72586 | 23 | 41 | 98 | 0 | 2 | 17 |
| Chr 7 | 3.7 | 38212 | 42702 | 72254 | 41 | 31 | 118 | 2 | 1 | 19 |
| Chr 8 | 3.0 | 27814 | 32272 | 73687 | 27 | 27 | 71 | 1 | 0 | 12 |
| Chr 9 | 2.5 | 19339 | 22797 | 33251 | 22 | 21 | 87 | 2 | 0 | 15 |
| Chr 10 | 2.2 | 15648 | 17563 | 31757 | 22 | 17 | 75 | 3 | 0 | 11 |
| Chr 11 | 2.1 | 19302 | 21735 | 37843 | 21 | 16 | 62 | 2 | 0 | 11 |
| Chr 12 | 2.0 | 17124 | 18059 | 31426 | 18 | 19 | 85 | 0 | 2 | 18 |
| Chr 13 | 1.8 | 13521 | 14955 | 25833 | 14 | 22 | 66 | 0 | 0 | 16 |
| Chr 14 | 1.5 | 11497 | 13115 | 15375 | 7 | 16 | 72 | 0 | 0 | 10 |
| Chr 15 | 1.3 | 11117 | 11845 | 14766 | 9 | 18 | 44 | 0 | 0 | 9 |
| Chr 16 | 0.0 | 276 | 274 | 365 | 0 | 0 | 0 | 0 | 0 | 0 |
| Chr 17 | 1.1 | 6718 | 7975 | 11845 | 13 | 39 | 23 | 1 | 1 | 5 |
| Chr 18 | 1.1 | 4424 | 5190 | 9316 | 8 | 10 | 27 | 1 | 1 | 4 |
| Chr 19 | 1.0 | 6830 | 7990 | 9351 | 12 | 14 | 30 | 1 | 1 | 5 |
| Chr 20 | 1.4 | 13686 | 17524 | 21656 | 18 | 30 | 63 | 1 | 3 | 14 |
| Chr 21 | 0.7 | 5578 | 6922 | 5921 | 3 | 9 | 23 | 0 | 0 | 5 |
| Chr 22 | 0.4 | 1404 | 1336 | 2997 | 7 | 6 | 8 | 0 | 0 | 1 |
| Chr 23 | 0.6 | 4026 | 5036 | 4772 | 8 | 10 | 36 | 0 | 2 | 4 |
| Chr 24 | 0.6 | 3568 | 4589 | 8636 | 1 | 18 | 20 | 0 | 2 | 2 |
| Chr 25 | 0.2 | 976 | 1365 | 1607 | 1 | 4 | 3 | 0 | 0 | 0 |
| Chr 26 | 0.5 | 3820 | 4430 | 3682 | 1 | 5 | 24 | 0 | 0 | 8 |
| Chr 27 | 0.5 | 4193 | 5116 | 7415 | 3 | 6 | 10 | 0 | 0 | 0 |
| Chr 28 | 0.4 | 4320 | 5599 | 5990 | 0 | 2 | 8 | 0 | 0 | 0 |
| Chr E22 | 0.1 | 335 | 599 | 307 | 0 | 0 | 3 | 0 | 0 | 0 |
| Chr E64 | 0.0 | 46 | 46 | 155 | 0 | 0 | 0 | 0 | 0 | 0 |
| Chr W | 0.0 | 176 | 153 | 213 | 0 | 0 | 1 | 0 | 0 | 0 |
| Chr Z | 7.2 | 108527 | 133615 | 283727 | 94 | 69 | 179 | 0 | 0 | 25 |
| Total | 100.0 | 1163685 | 1379196 | 2842291 | 1059 | 1149 | 2934 | 34 | 24 | 479 |

## Supplementary Table 3. Wald’s tests of the distribution of hits among chicken chromosomes for various subsets of the data.

Values above 2 indicate significant overrepresentation and values under -2 indicate significant underrepresentation on a given chicken chromosome listed at left. n/a indicates Wald’s test could not be calculated due to an observed value of 0.

|  |  | Value of Wald’s test | | | | | | | | |
| --- | --- | --- | --- | --- | --- | --- | --- | --- | --- | --- |
|  |  | All hits for clones with one significant sequence | | | Single best hit for clones with one significant sequence | | | Clones with high quality paired hits | | |
| Chicken chromosome | Percentage of chicken genome | Alligator | Turtle | Emu | Alligator | Turtle | Emu | Alligator | Turtle | Emu |
| Chr 1 | 19.5 | 142.4 | 167.2 | 317.5 | 5.3 | 2.6 | 0.0 | -0.3 | -2.1 | -1.4 |
| Chr 2 | 15.0 | 67.8 | 81.9 | 170.6 | -2.5 | -1.1 | -1.9 | -1.3 | -2.8 | 0.0 |
| Chr 3 | 11.0 | 6.1 | 10.0 | 69.8 | 0.4 | -0.2 | 0.1 | -1.3 | -0.5 | 0.3 |
| Chr 4 | 9.1 | 2.8 | -10.3 | 37.3 | 0.1 | -1.9 | -1.4 | 0.9 | -0.1 | -1.7 |
| Chr 5 | 6.0 | -33.0 | -42.7 | -91.4 | -2.4 | -0.3 | -2.3 | 1.0 | 0.4 | -0.8 |
| Chr 6 | 3.6 | -37.8 | -44.5 | -96.3 | -3.3 | -0.1 | -0.8 | n/a | 0.8 | -0.1 |
| Chr 7 | 3.7 | -24.7 | -38.5 | -104.6 | 0.3 | -2.1 | 0.8 | 0.5 | 0.1 | 0.3 |
| Chr 8 | 3.0 | -36.8 | -43.6 | -37.5 | -0.9 | -1.4 | -1.9 | 0.0 | n/a | -0.7 |
| Chr 9 | 2.5 | -56.4 | -62.1 | -141.5 | -0.9 | -1.6 | 1.6 | 0.8 | n/a | 0.8 |
| Chr 10 | 2.2 | -61.9 | -73.2 | -123.0 | -0.2 | -2.0 | 1.3 | 1.3 | n/a | 0.2 |
| Chr 11 | 2.1 | -34.7 | -44.6 | -92.6 | -0.3 | -2.1 | 0.0 | 0.9 | n/a | 0.3 |
| Chr 12 | 2.0 | -39.9 | -57.1 | -106.6 | -0.7 | -0.9 | 2.9 | n/a | 1.1 | 2.0 |
| Chr 13 | 1.8 | -53.8 | -65.4 | -116.0 | -1.5 | 0.2 | 1.5 | n/a | n/a | 1.8 |
| Chr 14 | 1.5 | -47.8 | -55.6 | -136.0 | -3.5 | -0.4 | 3.2 | n/a | n/a | 0.8 |
| Chr 15 | 1.3 | -29.1 | -41.9 | -111.5 | -1.4 | 0.8 | 1.1 | n/a | n/a | 1.0 |
| Chr 16 | 0.0 | -9.6 | -12.7 | -24.0 | n/a | n/a | n/a | n/a | n/a | n/a |
| Chr 17 | 1.1 | -52.7 | -57.3 | -108.5 | 0.4 | 4.3 | -1.8 | 0.6 | 0.7 | -0.1 |
| Chr 18 | 1.1 | -71.5 | -78.2 | -120.3 | -1.1 | -0.7 | -0.8 | 0.6 | 0.8 | -0.5 |
| Chr 19 | 1.0 | -41.5 | -46.0 | -109.3 | 0.5 | 0.8 | 0.3 | 0.7 | 0.8 | 0.2 |
| Chr 20 | 1.4 | -16.6 | -8.5 | -86.4 | 0.9 | 2.7 | 3.0 | 0.5 | 1.6 | 2.0 |
| Chr 21 | 0.7 | -25.7 | -24.7 | -96.0 | -2.4 | 0.4 | 0.7 | n/a | n/a | 0.8 |
| Chr 22 | 0.4 | -45.6 | -54.2 | -75.4 | 1.1 | 0.7 | -1.1 | n/a | n/a | -0.8 |
| Chr 23 | 0.6 | -33.8 | -33.9 | -92.2 | 0.6 | 1.0 | 3.1 | n/a | 1.3 | 0.6 |
| Chr 24 | 0.6 | -43.1 | -43.0 | -67.9 | -5.6 | 2.6 | 0.4 | n/a | 1.3 | -0.7 |
| Chr 25 | 0.2 | -27.5 | -26.0 | -53.4 | -1.1 | 0.9 | -1.6 | n/a | n/a | n/a |
| Chr 26 | 0.5 | -25.5 | -28.9 | -87.6 | -4.2 | -0.3 | 1.9 | n/a | n/a | 2.0 |
| Chr 27 | 0.5 | -17.2 | -16.9 | -51.4 | -1.1 | 0.3 | -1.2 | n/a | n/a | n/a |
| Chr 28 | 0.4 | -10.8 | -5.5 | -57.8 | n/a | -2.1 | -1.7 | n/a | n/a | n/a |
| Chr E22 | 0.1 | -21.3 | -17.4 | -43.6 | n/a | n/a | 0.3 | n/a | n/a | n/a |
| Chr E64 | 0.0 | -1.6 | -2.8 | 1.1 | n/a | n/a | n/a | n/a | n/a | n/a |
| Chr W | 0.0 | -6.7 | -10.3 | -18.7 | n/a | n/a | 0.3 | n/a | n/a | n/a |
| Chr Z | 7.2 | 87.6 | 111.8 | 179.7 | 1.9 | -1.7 | -2.6 | n/a | n/a | -2.0 |

## Supplementary Table 4. Statistics of BAC clones with paired hits to the chicken genome.

|  | No. (percentage) of BAC clones with any paired hits | Total number of paired blast hits | Number (percentage) of clones with high quality blast hits | Average length of BLAST hits in paired hits | Average length of a BLAST hits for clones with single successful BLAST sequence |
| --- | --- | --- | --- | --- | --- |
| Alligator | 63 (3.8) | 22,881 | 34 (1.0) | 56.71 bp (±48.96) | 32.9 bp (±10.20) |
| Turtle | 60 (3.3) | 5,751 | 24 (0.7) | 71.86 bp (±77.66) | 33.6 bp (±12.16) |
| Emu | 545 (18.6) | 44,099 | 479 (16.3) | 115.68 bp (±93) | 30.0 bp (±13.20) |

Note: Three BAC clones with high quality paired hits from the Turtle were discarded because they mapped to sites virtually identical to those of other Turtle BAC clones. These BAC clones could be duplicated in the library or somehow made redundant during the sequencing process.

# Figures

## Figure 1. Distribution of lengths of BLAST hits for all significant hits


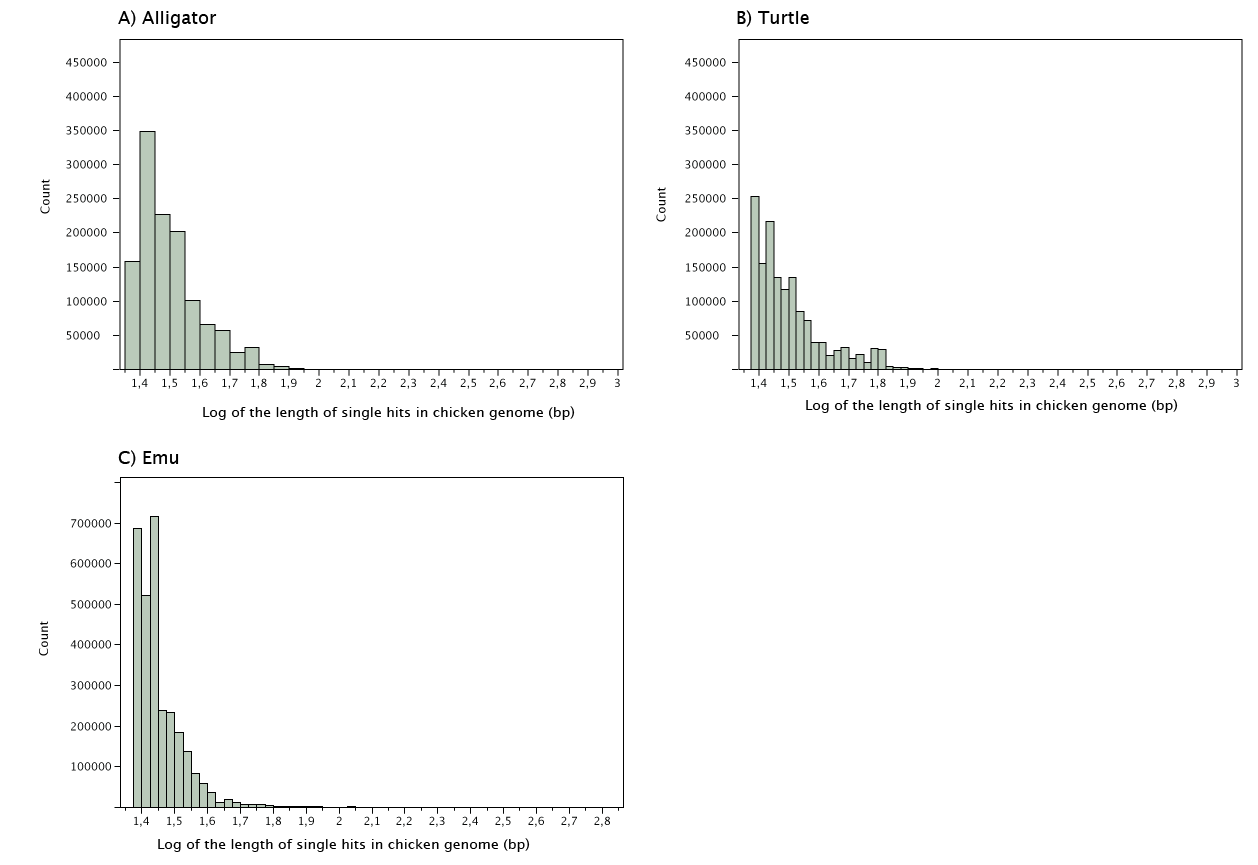


Figure 1. X-axis is log base 10. A, Alligator; B, Turtle, C, Emu.

## Figure 2. Distribution of percent identities for all hits.


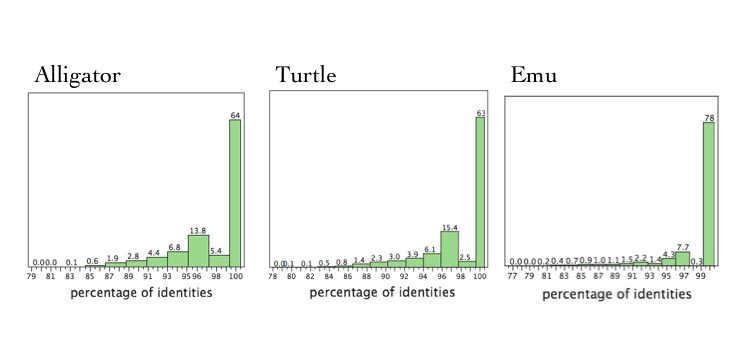


Figure 2. Numbers above bars are percentages.

## Figure 3. Distribution of high quality paired hits per chicken chromosome

Figure 3. On the x-axis are the different chicken chromosomes. On the Y-axis are the percentage of all high quality paired hits (Supplementary Table 2) for the three query species, and of the genome comprised by each chicken chromosome.

## Figure 4. Distribution of intermarker distances in the chicken genome for high quality paired hits for alligator, turtle and emu.


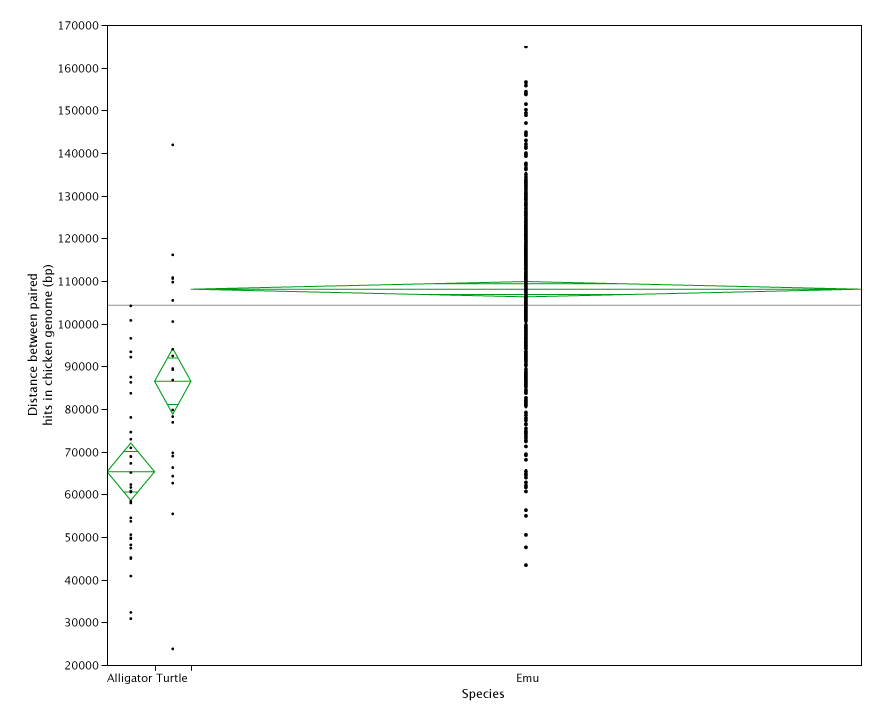
Figure 4. For each species the green diamond shape corresponds to the mean and the standard deviation. The Student’s t-test, ANOVA and Van de Waerden tests show that the distributions are different.

## Correlation between the number of high quality paired hits for emu and size of the target chicken chromosome

R^2^ = 0.965, P < 0.0001.


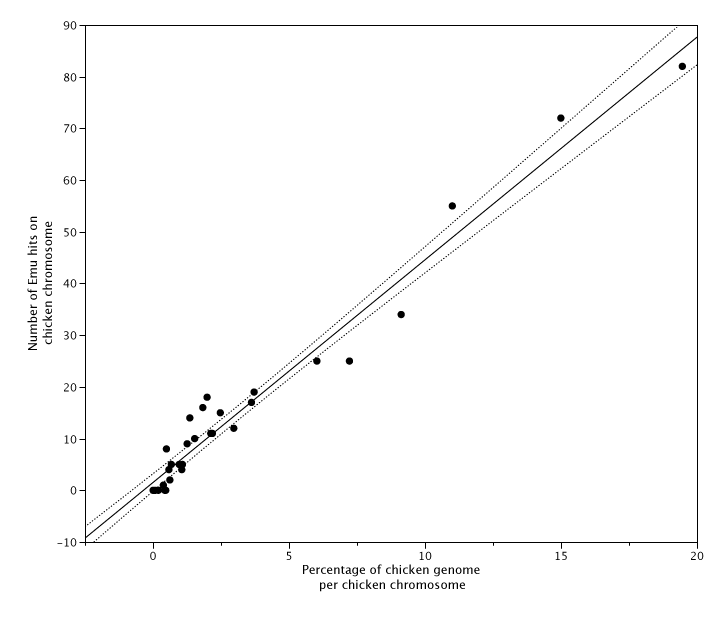


## Supplementary spreadsheet listing inferred gene contents of BACs with high quality paired hits

The Excel spreadsheet is included as a separate file “Chapus-Edwards-gene-content.xls”. It lists the inferred gene contents of all BACs yielding high quality paired hits using methods described in the main text. The table is composed of three tabs (one for each species). Each row represents a high quality paired hit. The columns list the information about each BAC clone and its associated blast hits. For the Alligator and the Turtle, each BAC clone is represented by its genbank ID. The Emu BAC clones are indicated by the plate number in the library and their well in specific 384-well plates. The other columns list information on: the number of hits of each BAC clone to chicken genome, the length of these hits, the positions of the hits on the chicken genome, the e-value/length/position of the blast hit for the forward (**F**) and reverse (**R**) ends. The comments column indicates the gene content on the chicken genome between the two ends. The “link pdf” column gives the name of the corresponding PDF file listing screenshots from the UCSC Genome Browser of the gene content, genome conservation among vertebrates between the two ends. These pdfs are available upon request. For the Emu tab the shaded rows indicate to BAC clones selected for fingerprinting. In the Turtle tab, shaded rows indicate BAC clones with very similar if not identical BLAST locations corresponding to the same chicken mapped position and same blast hits.
